# Challenges and opportunities of catalytic decomposition of ozone at room temperature

**DOI:** 10.1016/j.fmre.2025.12.001

**Published:** 2025-12-09

**Authors:** Xiaotong Li, Zhisheng Wang, Biwu Chu, Jinzhu Ma, Hong He

**Affiliations:** a State Key Joint Laboratory of Environment Simulation and Pollution Control, Research Center for Eco-Environmental Sciences, Chinese Academy of Sciences, Beijing 100085, China; b Center for Excellence in Regional Atmospheric Environment, Institute of Urban Environment, Chinese Academy of Sciences, Xiamen 361021, China; c College of Resources and Environment, University of Chinese Academy of Sciences, Beijing 100049, China

**Keywords:** Environmental catalysis, Ozone decomposition, Manganese oxides, Ag-based catalysts, Layered double hydroxide (LDH)

## Abstract

Catalytic decomposition is a key technology for the removal of ozone (O_3_). Practical application scenarios require catalysts with superior O_3_ decomposition performance at ambient temperatures, high humidity, and high space velocities. In this review, the strategies for the design of catalysts with efficient and stable O_3_ decomposition performance are presented, and the three factors that limit the catalytic decomposition of O_3_ at ambient temperature (insufficient active sites, competitive adsorption of H_2_O and O_3_ molecules, and difficult desorption of intermediate oxygen species) are systematically summarized for the first time. Subsequently, the research progress in recent years is categorized and summarized in terms of addressing the three factors limiting O_3_ decomposition, which in turn suggests the shortcomings of current research and the focus of future research.

## Graphical abstract

**Figure:**
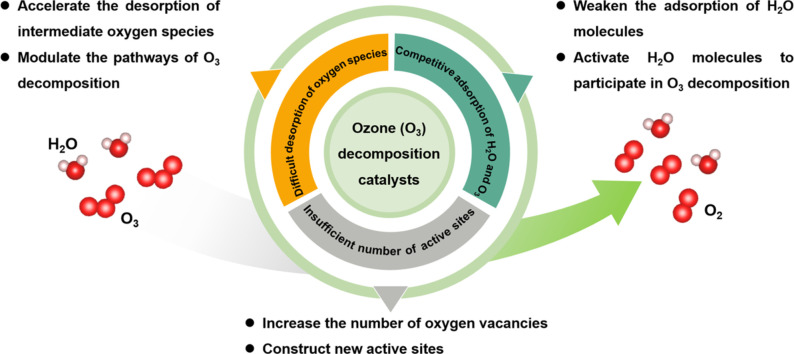

## 1 Introduction

Ozone (O_3_), as a strong oxidizing agent, affects the oxidizing capacity of the atmosphere and the formation of secondary particulate matter [1]. In addition, O_3_ is also harmful to ecosystems and human health [[2], [3], [4]]. In addition to the guideline value of 100 µg/m^3^ (∼51 ppb) for the 8-h average O_3_ concentration, the World Health Organization introduced a peak seasonal average O_3_ concentration of 60 µg/m^3^ (∼31 ppb) in 2021 based on the effects of long-term O_3_ exposure on the total mortality rate and the mortality rate from respiratory diseases. The standard heat, standard free energy and entropy of the O_3_ decomposition reaction are −142 kJ/mol, −163 kJ/mol and 239 J/(K·mol), respectively, indicating that O_3_ is thermodynamically unstable and can spontaneously decompose to oxygen [5]. At atmospheric pressure, the half-life of O_3_ in oxygen at 25 °C, 100 °C and 250 °C is 160 h, 210 s and 0.03 s, respectively [5]. Thus, O_3_ is stable at room temperature. Catalysts can lower the energy barrier of O_3_ decomposition and serve to accelerate the decomposition of O_3_.

Highly efficient catalytic decomposition of O_3_ is urgently needed for treating industrial exhaust and purifying air in confined spaces and atmospheric environments. During the treatment of flue gas, wastewater and drinking water by the catalytic ozonation technology, excess O_3_ is injected to achieve higher removal efficiencies, and practical application conditions are variable, which can lead to O_3_ residues (80 ppb–70 ppm) in the exhaust [[5], [6], [7]]. Catalysts with efficient and stable activity at ambient temperature (20−70 °C), high humidity and medium space velocity need to be developed for the removal of residual O_3_ in the exhaust. In addition, the exhausts contain other pollutants such as NO*_x_*, SO_2_ and VOCs, so it is necessary to develop catalysts with high resistance to poisoning. O_3_ pollution (50–300 ppb) is also present in the indoor environments due to the operation of disinfection equipment, negative ion generators, electrostatic precipitators, photocopiers and laser-jet printers [8,9]. Catalysts with efficient and stable activity at room temperature, moderate humidity, and high space velocity need to be developed for the removal of O_3_ in indoor environments. The cabins of aircraft flying at high altitudes also face O_3_ pollution (1800 ppb) [10,11]. Catalysts with efficient and stable activity at low temperature, low humidity and ultra-high space velocity must be developed to remove O_3_ in the cabins [[11], [12], [13], [14], [15]].

In the atmosphere, O_3_ is a typical secondary pollutant. The relationship between O_3_ concentration and its precursors (NO*_x_* and VOCs) is non-linear [1,3,16]. In an urban environment, O_3_ is usually more sensitive to VOCs, which are difficult to control due to their highly complex and dispersed emissions [17]. Moreover, O_3_ pollution may even be exacerbated by the rapid reduction of NO*_x_* [17]. As a result, effectively solving the O_3_ pollution by reducing the precursors from the emission sources remains a great challenge in many countries. In this case, catalytic decomposition of O_3_ has become a potential ancillary means of solving O_3_ pollution in the atmospheric environment. Catalysts with efficient and stable activity at room/low temperature, medium to high humidity, and ultra-high space velocity need to be developed for the removal of O_3_ in atmospheric environments. In addition, complex atmospheric environments, such as co-existing pollutants, place even greater demands on the catalyst. Therefore, the development of catalysts with efficient and stable activity for O_3_ decomposition in various scenarios is the key to practical applications.

## 2 Optimization strategies of catalysts based on the main factors limiting O_3_ decomposition

Among various catalysts for the decomposition of O_3_, manganese oxides (MnO*_x_*) have attracted much attention due to their excellent activity and low cost. The active site for the decomposition of O_3_ on MnO*_x_* catalysts is oxygen vacancies (V_O_) [18,19]. The detailed mechanism of O_3_ decomposition is described below (Eqs. (1)–(3)) [20,21]:

(1) $$O_{3}+V_{O}-Mn^{n+}\to O_{2}+O^{2-}-Mn^{\left(n+2\right)+}\left(n\;\text{represents}\;2,3\;\text{or}\;4\right)$$

(2) $$O_{3}+O^{2-}-Mn^{\left(n+2\right)+}\to O_{2}+{O_{2}}^{2-}-Mn^{\left(n+2\right)+}$$

(3) $${O_{2}}^{2-}-Mn^{\left(n+2\right)+}\to O_{2}+V_{O}-Mn^{n+}$$

**Fig. 1:**
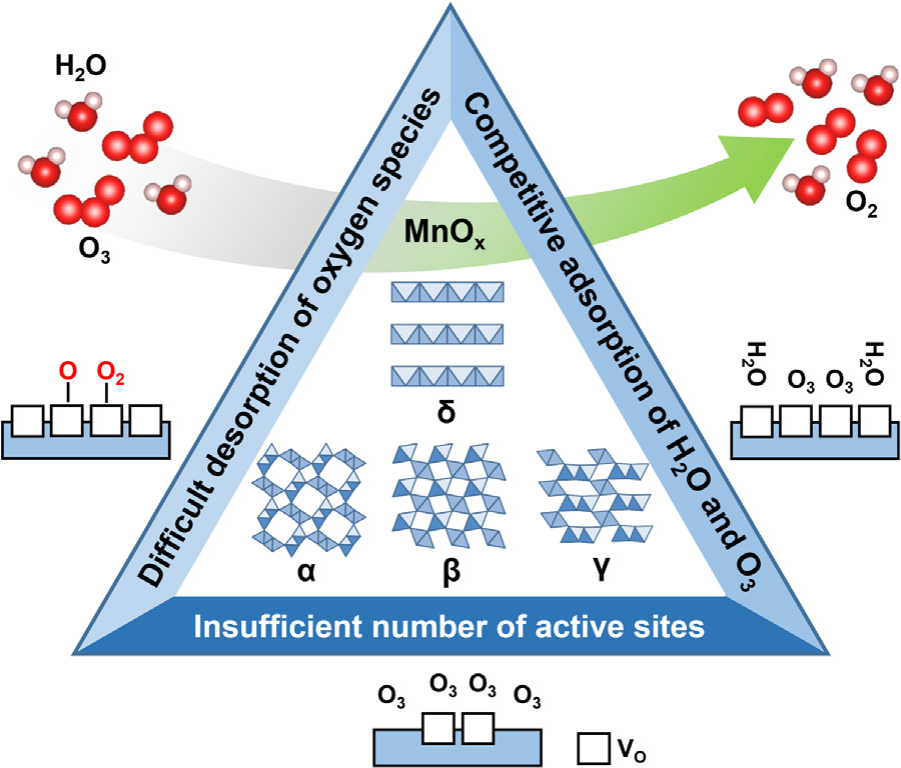
**The key factors limiting O_3_ decomposition on MnO*_x_* catalysts**.

Oxygen vacancies act as electron donors and acceptors for redox reactions during the adsorption of O_3_ and the desorption of intermediate oxygen species, respectively. By applying the methodology of classifying and summarizing the limiting factors of performance in the preliminary study [22,23], the limiting factors about the activity of MnO*_x_* catalysts for the decomposition of O_3_ were identified. Firstly, the insufficient number of oxygen vacancies will limit the activity of MnO*_x_* catalysts for the decomposition of O_3_ (Fig. 1) [24,25]. In addition, the decomposition of O_3_ is inevitably confronted with the competitive adsorption of H_2_O and O_3_ molecules (Fig. 1) [25]. Finally, in terms of the O_3_ decomposition mechanism, the desorption of peroxide species in the final step is a rate-limiting step, so the difficult desorption of peroxide species is another important factor limiting the decomposition of O_3_ (Fig. 1) [19,20,26]. In summary, the key factors limiting the decomposition of O_3_ are the insufficient number of active sites, the competitive adsorption of H_2_O and O_3_ molecules, and the difficult desorption of intermediate oxygen species. Among them, the competitive adsorption of H_2_O and O_3_ molecules and the difficult desorption of intermediate oxygen species are the difficulties of O_3_ decomposition. The competitive adsorption of H_2_O and O_3_ molecules has a more rapid but reversible effect on the O_3_ decomposition activity of catalysts. The O_3_ decomposition activity of the catalyst usually decreases rapidly under transition from dry to humid conditions, indicating that H_2_O molecules have a significant effect on its activity. However, the O_3_ decomposition activity of the catalyst recovers rapidly when switching to dry conditions, indicating that the effect of H_2_O molecules on the O_3_ decomposition activity is reversible. Notably, H_2_O molecules are inevitably present in the actual environment, so the competitive adsorption of H_2_O and O_3_ molecules has a great influence on O_3_ decomposition activity. The effect of accumulated intermediate oxygen species on the O_3_ decomposition activity of catalysts is slow but difficult to recover. During O_3_ decomposition, intermediate oxygen species slowly accumulate on the catalysts, resulting in a gradual decline in their activity. To restore the activity after deactivation caused by the accumulation of intermediate oxygen species, high-temperature calcination is often necessary. In summary, the difficult desorption of intermediate oxygen species has a significant impact on O_3_ decomposition activity. Corresponding solutions to the above problems include increasing the number of active sites, weakening the adsorption of H_2_O molecules or activating H_2_O molecules to promote the participation of H_2_O molecules in the decomposition of O_3_, accelerating the desorption of intermediate oxygen species or modulating the pathway of O_3_ decomposition, respectively.

### 2.1 Increasing the number of active sites

**Fig. 2:**
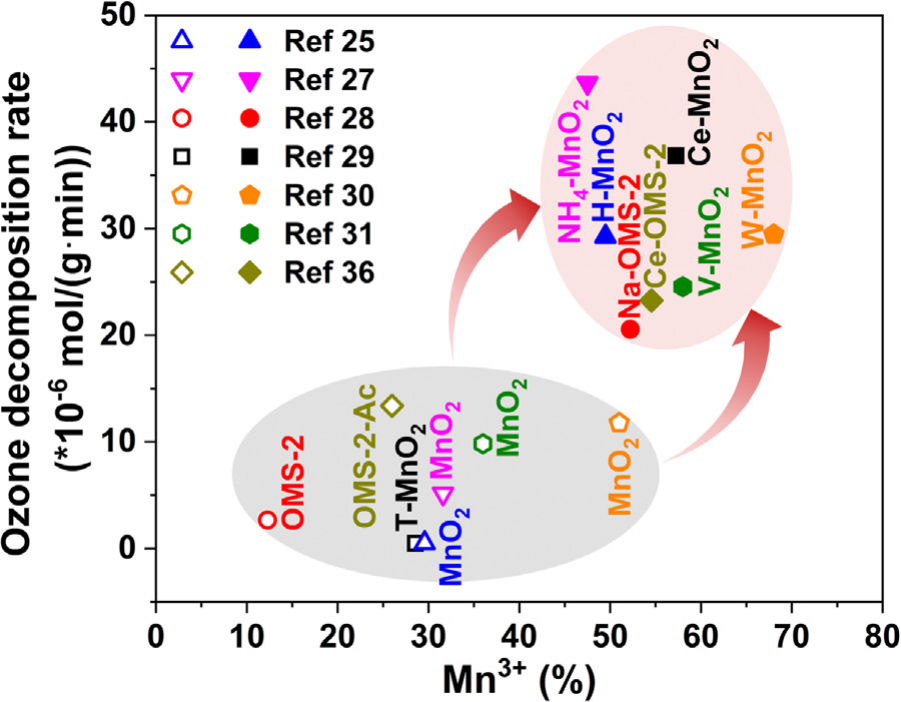
**The relationship between O_3_ decomposition rate and the number of Mn^3+^ (oxygen vacancies) in MnO_2_ catalysts** [28,30–34,39].

In previous studies, it has been determined that MnO_2_ catalysts have higher O_3_ decomposition activity than other MnO*_x_* (e.g., Mn_2_O_3_, Mn_3_O_4_ or MnO). Therefore, researchers have focused on regulating MnO_2_ catalysts, resulting in a series of highly efficient O_3_ decomposition catalysts. The O_3_ decomposition activity of manganese dioxides (MnO_2_) can be successfully improved by increasing the number of oxygen vacancies. As shown in Fig. 2, the number of oxygen vacancies in the modulated MnO_2_ is significantly increased compared to pristine MnO_2_, with a corresponding significant improvement in O_3_ decomposition activity. The main methods to increase the number of oxygen vacancies over MnO_2_ include modulation of the content and type of ions (H^+^, K^+^, NH_4_^+^ or Na^+^) in the tunnels of MnO_2_ [[27], [28], [29], [30], [31]], in situ doping of other metal elements (Ce, Fe, W or V) during synthesis [24,25,[32], [33], [34], [35]], vacuum deoxidation to remove oxygen atoms from MnO_2_ [36], or optimization of raw materials, conditions and methods of preparation [[37], [38], [39]]. Our group found that acetate groups can prevent the aggregation of MnO_2_ particles, thereby increasing the specific surface area and the number of oxygen vacancies on cryptomelane-type manganese oxide (OMS-2) catalysts [18]. The number of oxygen vacancies was further increased by in situ doping with Ce during the hydrothermal synthesis. Cerium-doped OMS-2 (Ce-OMS-2) catalyst showed O_3_ conversion of 90% at RH of 90% and space velocity of 600,000 h^−1^, which is a promising catalyst for the decomposition of O_3_ under high humidity [24]. Then, the hydrothermal conditions (fill percentage, hydrothermal temperature, and hydrothermal time) of the Ce-OMS-2 catalysts are further optimized to increase the number of oxygen vacancies and the O_3_ decomposition activity of the catalyst, and the Ce-OMS-2 catalysts that prepared under 80% of fill percentage, 95–100 °C of hydrothermal temperature and 8–24 h of hydrothermal time showed higher O_3_ conversion than other Ce-OMS-2 catalysts [38,39].

**Fig. 3:**
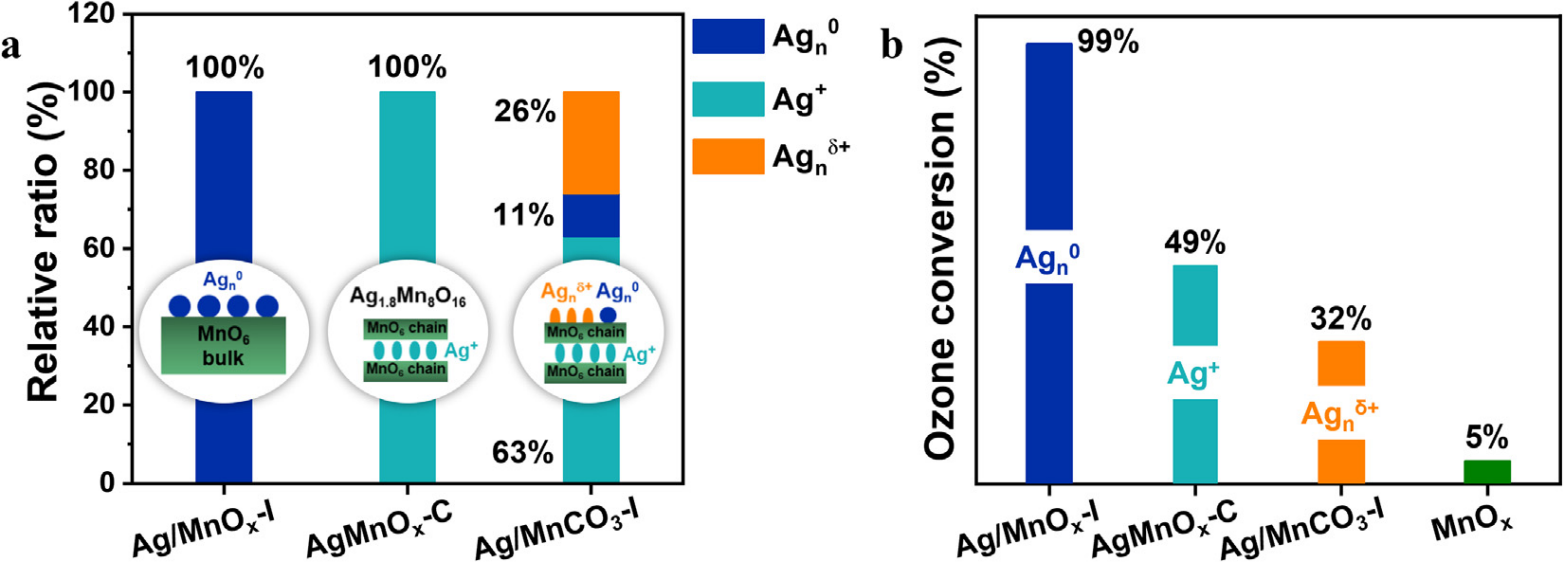
(a) Schematic diagram and relative ratio of Ag species on Ag-Mn catalysts. (b) The relationship between O_3_ decomposition activity (Conditions: ozone inlet concentration 40 ppm, temperature 30 °C, relative humidity 65%, and space velocity 840 L g^−1^ h^−1^) and the state of Ag species [40].

In response to the insufficient number of oxygen vacancies on MnO*_x_* catalysts, Ag active sites have been constructed to improve the O_3_ decomposition performance of MnO*_x_* catalysts [40]. Under a relative humidity of 65% and a high space velocity of 840,000 h^−1^, the 6%Ag/MnO*_x_*-I catalyst prepared by the impregnation method showed a 99% conversion of 40 ppm O_3_ after 6 h, which was far superior to the Ag-Mn catalysts prepared by other methods and the pure MnO*_x_* support (Fig. 3) [40]. The excellent O_3_ decomposition performance of the 6%Ag/MnO*_x_*-I catalyst is attributed to the formation of metallic Ag nanoparticles (Ag_n_^0^) on it. Ag_n_^0^ was determined to be a better active site for the decomposition of O_3_ than Ag_1.8_Mn_8_O_16_ and oxidized Ag clusters (Ag_n_^δ+^) [40,41], which is attributed to the weaker competitive adsorption of H_2_O and O_3_ molecules, faster activation of O_3_ molecules, and easier desorption of O_2_^2-^ [42]. More profoundly, the excellent activity of Ag_n_^0^ stems from the highly efficient electron transfer between Ag_n_^0^ and O_3_ molecules. The decomposition of O_3_ on Ag_n_^0^ is a redox reaction (Eqs. (4)–(6)):

(4) $$O_{3}+A{g_{n}}^{0}\to O_{2}+O^{2-}A{g_{n}}^{2+}$$

(5) $$O_{3}+O^{2-}A{g_{n}}^{2+}\to O_{2}+{O_{2}}^{2-}A{g_{n}}^{2+}$$

(6) $${O_{2}}^{2-}A{g_{n}}^{2+}\to O_{2}+A{g_{n}}^{0}$$

(7) $${O_{2}}^{2-}A{g_{n}}^{2+}\to Ag_{2}O_{2}+A{g_{n-2}}^{0}$$

Then, to address the low utilization of Ag due to large Ag nanoparticles and uneven distribution of Ag nanoparticles on the current catalysts, abundant manganese vacancies are constructed by modulating the morphology of MnO*_x_* supports to enhance the interaction between Ag and MnO*_x_* supports; finally Ag_n_^0^ is uniformly dispersed and more metallic Ag sites are exposed [43]. The excellent O_3_ decomposition performance was achieved with a lower Ag loading (2%) on the Ag/MnO*_x_*-C catalysts (catalysts with Ag supported on porous cubic MnO*_x_*), which was only one third of the Ag loading of the previously reported Ag/MnO*_x_* catalyst [43].

### 2.2 Weakening the adsorption of H_2_O molecules or activating H_2_O molecules to participate in the decomposition of O_3_

**Fig. 4:**
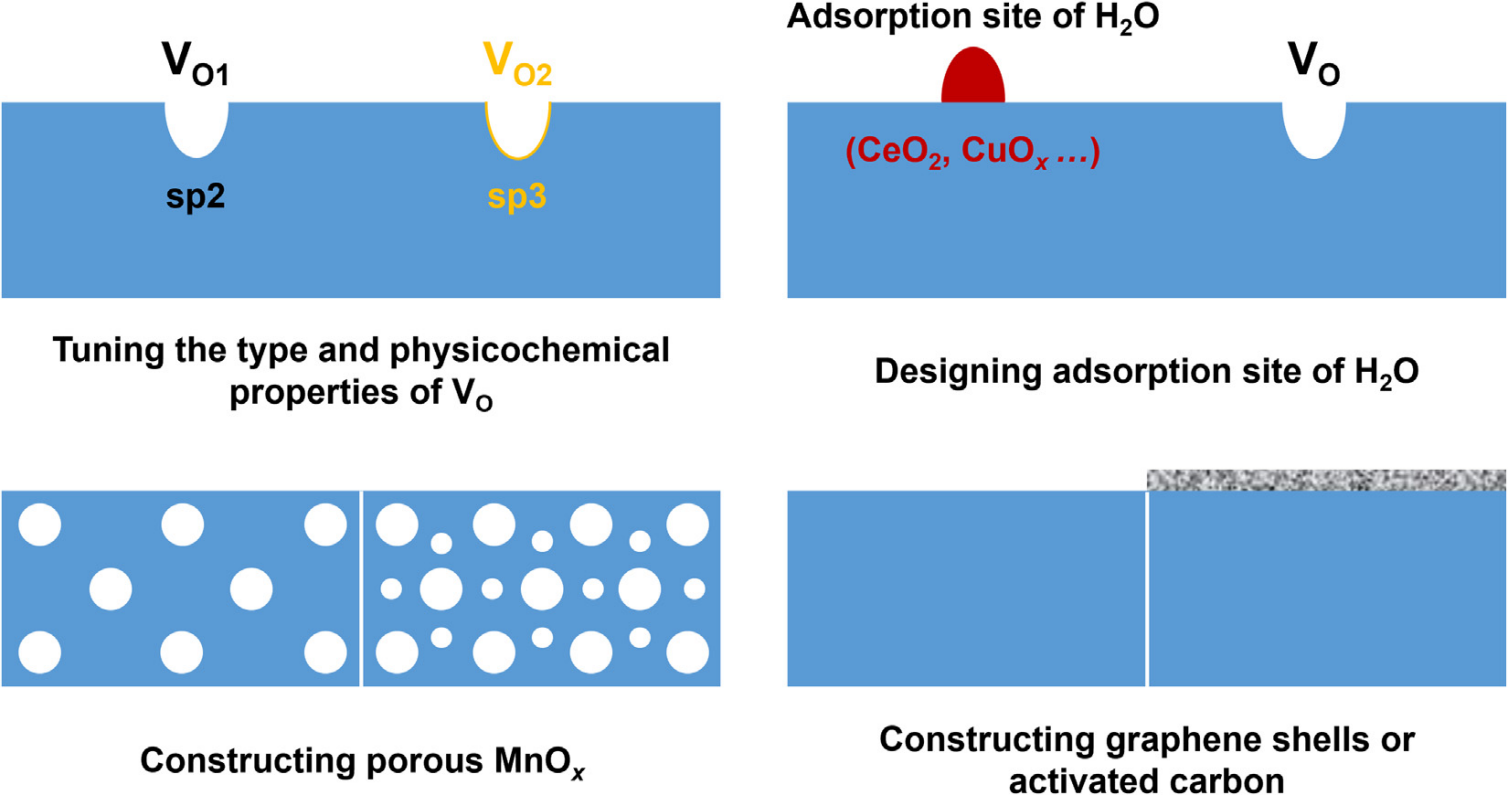
**Measures to alleviate the adsorption of H_2_O molecules on manganese oxides** [44–55].

**Fig. 5:**
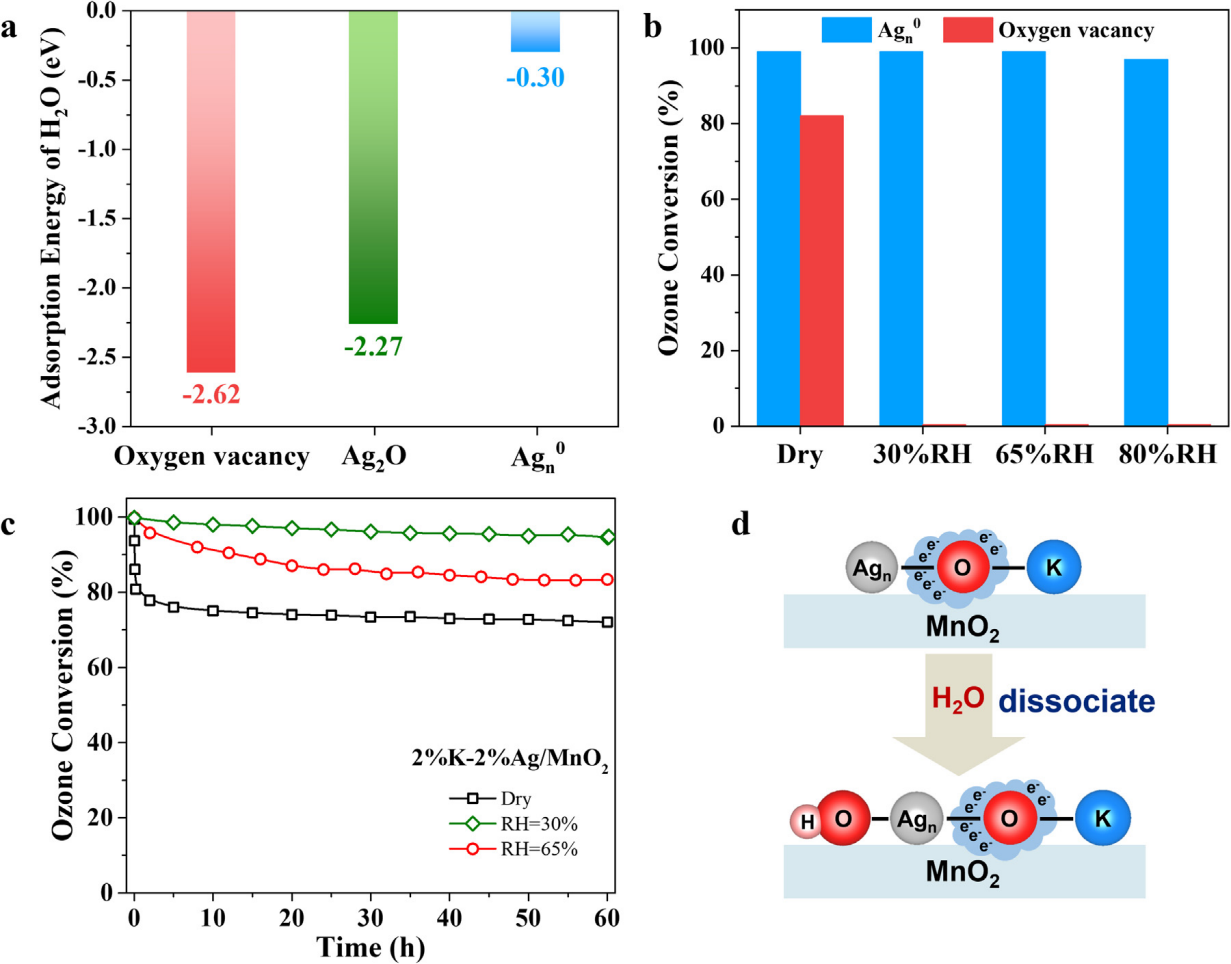
(a) Adsorption energy of H_2_O on Ag_n_^0^, Ag_2_O and oxygen vacancy of the MnO*_x_*. (b) O_3_ decomposition activity of Ag_n_^0^ and oxygen vacancy under dry gas, relative humidity of 30%, 65% and 80%. (c) O_3_ decomposition activity of K-Ag/MnO*_x_* catalyst under dry gas, relative humidity of 30% and 65%. (d) The formation mechanism of hydroxylated Ag active sites [40,44,56]. Conditions: ozone inlet concentration 40 ppm, temperature 30 °C, and space velocity 840 L g^−1^ h^−1^.

The adsorption of H_2_O molecules on manganese oxides can be alleviated to some extent by modulating the type and physicochemical properties of oxygen vacancies [[44], [45], [46]], designing non-oxygen vacancy sites for the adsorption of H_2_O molecules [[47], [48], [49]], constructing porous manganese oxides with abundant macropores and mesopores [50,51], constructing isolated hydrophilic sites (N-Mn-N) over TpBpy-COF catalyst [52], and combining hydrophobic graphene shells [53], functionalized activated carbon [49,54] and hydrophobic carbon layer [55] with manganese oxides (Fig. 4). Besides, DFT calculations revealed that the adsorption of H_2_O molecules on the metallic Ag sites was much weaker than their adsorption on the oxygen vacancies of MnO*_x_* (Fig. 5a), so the competitive adsorption of H_2_O and O_3_ molecules can be attenuated by constructing metallic Ag sites on MnO*_x_*. When the relative humidity increased to 30%, the MnO*_x_* catalyst had no O_3_ decomposition activity due to the competitive adsorption of H_2_O and O_3_ molecules (Fig. 5b). However, when the relative humidity increased to 80%, the 6%Ag/MnO*_x_* catalyst still retained 97% of the O_3_ conversion (Fig. 5b), indicating that the H_2_O resistance of the catalyst was significantly improved by the construction of metallic Ag sites [40].

**Fig. 6:**
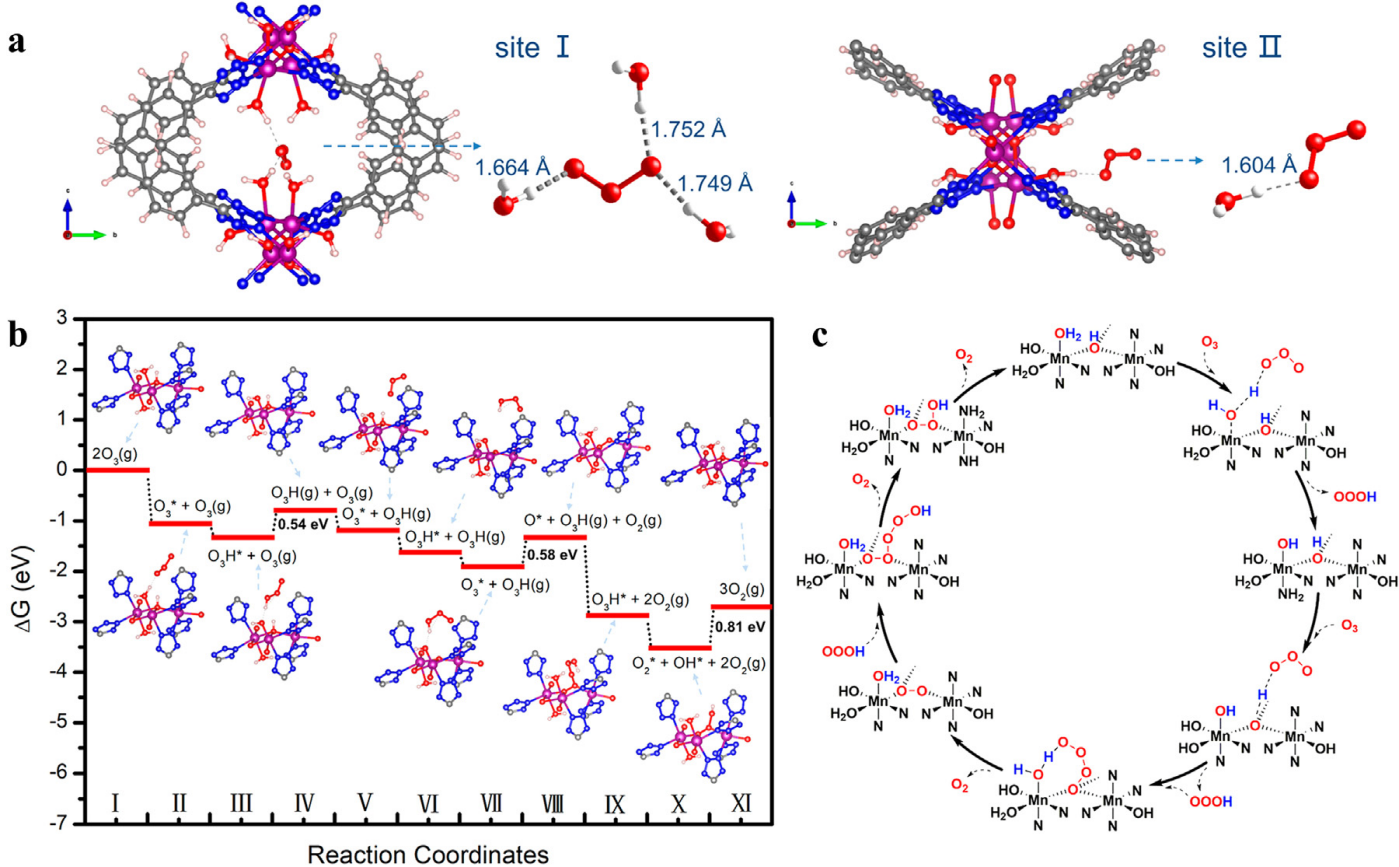
(a) Calculated O_3_ adsorption sites on ZZU-281. (b) Free energy diagrams and optimized structures intermediates along the ZZU-281-catalyzed O_3_ decomposition pathway. (c) Proposed mechanism of O_3_ degradation by ZZU-281 [58].

Activating H_2_O molecules to participate in the decomposition of O_3_ is also an efficient means of solving the competitive adsorption of H_2_O and O_3_ molecules. H_2_O molecules could enhance the O_3_ decomposition activity of K-Ag/MnO*_x_* catalysts (Fig. 5c), suggesting that the competitive adsorption of H_2_O and O_3_ molecules on Ag/MnO*_x_* catalysts was further resolved by the addition of alkali metals [56]. Due to electron transfer by alkali metals, the O atom with a more negative valence in Ag_2_O-K can take away H atoms of H_2_O molecules, favoring the adsorption and dissociation of H_2_O molecules to form the new hydroxylated Ag active sites (Ag-O(OH)*_x_*-K) with high activity for O_3_ decomposition (Fig. 5d). In summary, it is tentatively speculated that the common features of sites activating H_2_O molecules are the more negative valence of the O atom in metal oxides. On the new materials such as iron-organic framework (MIL-100(Fe)) [57], manganese-organic framework (ZZU-281(Mn)) [58], bimetallic-organic framework (PCN-250(Fe_2_Co)) [59] and monolithic MoO_3_/graphdiyne [60], the H_2_O molecules or OH groups activated during the humid O_3_ decomposition or present in the structure of the catalysts are active sites for O_3_ decomposition, and the key step is the transfer of the H atom from the active sites to the first O_3_ molecule to form the OOOH radical. Taking ZZU-281(Mn) as an example, the specific reaction mechanism is as follows (Fig. 6) [58]. After the formation of the Mn-OH and OOOH radical, the second O_3_ molecule adsorbs on the adjacent OH group, followed by the rearrangement of the intermediate. Accompanied by the release of an O_2_ molecule, the coordinated H_2_O molecule is restored, and a Mn-O-O-Mn intermediate is formed. Then, the Mn-O-O-Mn species react with the OOOH radical to form Mn-O-O(OH)-Mn and an O_2_ molecule. Finally, Mn-O-O(OH)-Mn further releases another O_2_ molecule and the adjacent OH group is regenerated. In summary, the highly efficient H_2_O-activated O_3_ decomposition mechanism on the above new materials ensures the good activity of the catalysts under humid conditions, thus solving the competitive adsorption of H_2_O and O_3_ molecules. However, there is a tendency for slow oxidation of the organic ligands of metal-organic frameworks (MOFs) during the decomposition of O_3_. The preparation of the MOFs is complicated, and their yield is low. Therefore, it remains to be investigated whether the MOFs are good O_3_ decomposition materials for practical applications.

### 2.3 Accelerating the desorption of intermediate oxygen species or modulating the pathway of O_3_ decomposition

**Fig. 7:**
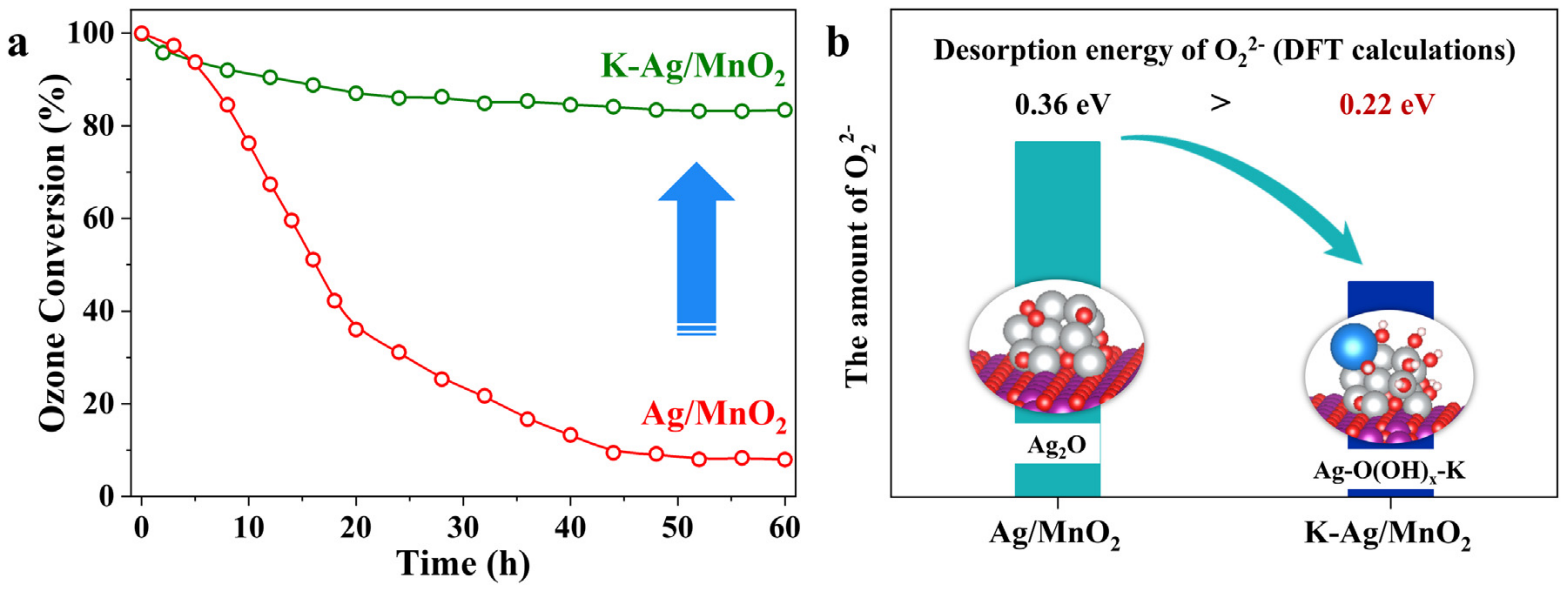
(a) O_3_ conversion (Conditions: ozone inlet concentration 40 ppm, temperature 30 °C, relative humidity 65%, and space velocity 840 L g^−1^ h^−1^) and (b) the amount and desorption energy of O_2_^2-^ on Ag/MnO*_x_* and K-Ag/MnO*_x_* catalysts [56].

To solve the difficult desorption of intermediate oxygen species on conventional MnO*_x_* catalysts during O_3_ decomposition, many measures have been taken to accelerate the capture of electron from intermediate oxygen species by the oxygen vacancies to rapidly release the active sites, such as the development of other Mn-based catalysts (mullite YMn_2_O_5_ [61], amorphous MnO*_x_* [62], spinel (Mn, Co)_3_O_4_ [63]), modulating the type of oxygen vacancies on MnO*_x_* [44,64], and modifying the physicochemical properties of oxygen vacancies by encapsulating MnO*_x_* with graphene layers [53], loading MnO*_x_* on functionalized activated carbon [49,54], coating hydrophobic carbon on mesocrystalline MnO [55], doping MnO*_x_* with chlorine during the synthesis [45], or synthesizing MnCO_3_/Mn_3_O_4_ heterostructure [65]. Although the competitive adsorption of H_2_O and O_3_ molecules has been solved by combining Ag and MnO*_x_*, the O_3_ decomposition on the metallic Ag sites is still a redox reaction. Metallic Ag sites are inevitably oxidized by intermediate oxygen species. To overcome the above problems, alkali metals were added to tune the electronic structure of the Ag active sites on the Ag/MnO*_x_* catalysts, promoting the dissociation of H_2_O molecules and forcing the Ag active sites to become hydroxylated [56]. The O_2_^2-^ species on the new active sites (Ag-O(OH)*_x_*-K) of the K-Ag/MnO*_x_* catalyst can easily desorb, thus significantly improving the O_3_ decomposition stability of the catalyst (Fig. 7) [56].

Modulating the pathways of O_3_ decomposition is an effective strategy to overcome the problems of O_3_ decomposition on metal oxides at room temperature. There are two pathways of O_3_ decomposition on metal oxides. In addition to oxygen vacancies, which can decompose ozone via the redox pathway, surface hydroxyl groups can also serve as active sites for the decomposition of O_3_ (Eq. (8)).

(8) $$\text{HO}-M-\text{OH}+O_{3}\to 2O_{2}+M-H_{2}O$$

**Fig. 8:**
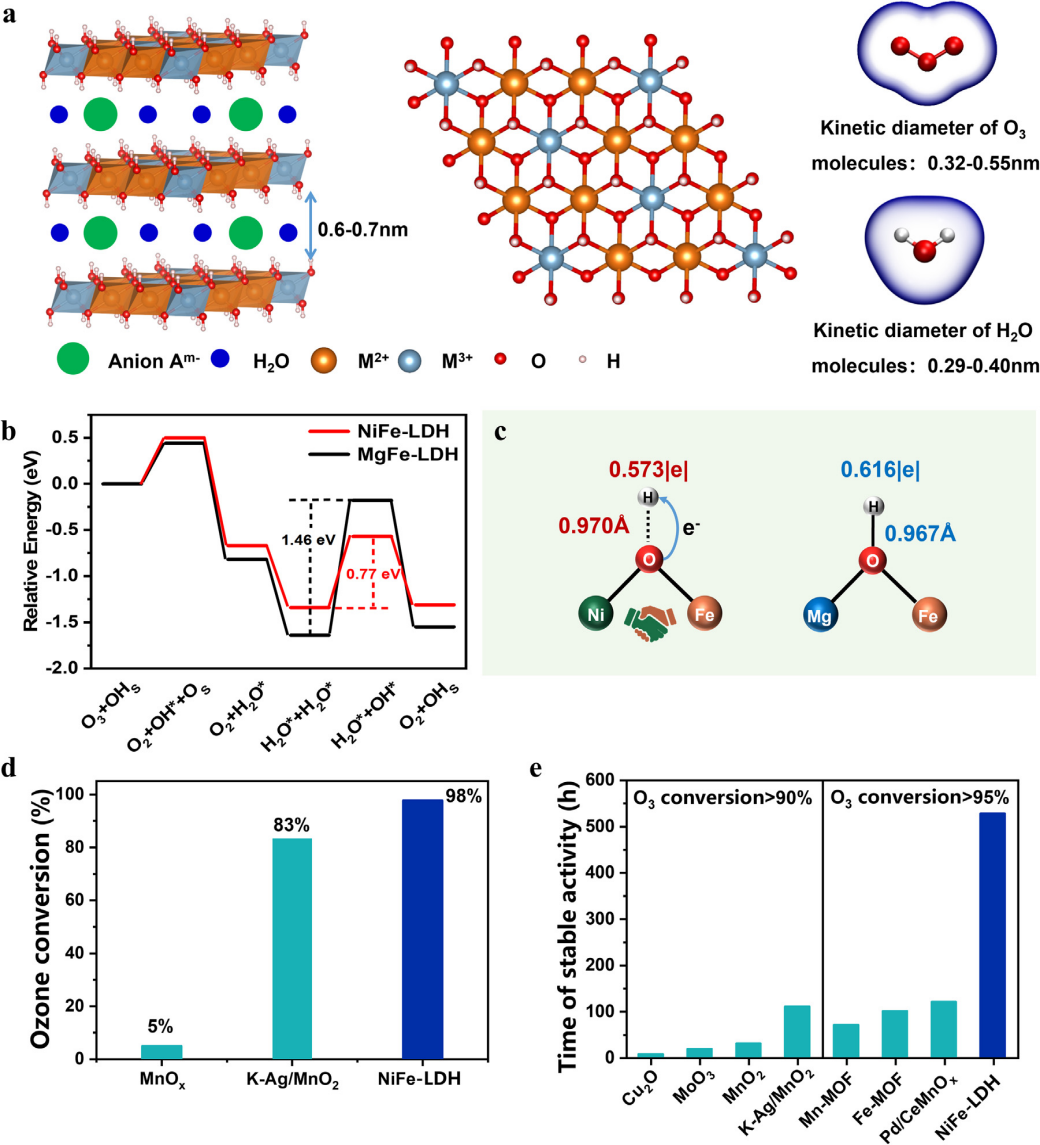
(a) Schematic diagram, (b-c) reaction pathway of LDH structure; (d-e) O_3_ decomposition activity and stability (Conditions: ozone inlet concentration 40 ppm, temperature 30 °C, relative humidity 65%, and space velocity 840 L g^−1^ h^−1^) of NiFe-LDH and other catalysts [56–58,60,67,–70,[72], [73]–74].

However, the number of hydroxyl groups on metal oxides is low, and the accumulation of reaction product H_2_O molecules on active sites will cause the deactivation of metal oxides [66]. The key to overcoming these problems lies in the activation of H_2_O molecules and the regeneration of hydroxyl groups. Layered Double Hydroxides (LDHs) are a two-dimensional layered material with suitable interlayer spacing, which facilitates the diffusion and reaction of O_3_ and H_2_O molecules (Fig. 8a) [67]. The abundant hydroxyl groups between the layers of LDHs can act as active sites for the decomposition of O_3_ [[67], [68], [69]]. In addition, the interlayer structure of LDHs can restrict H_2_O molecules, and the hydrogen bonding of hydroxyl groups between the interlayer structure of LDHs can activate H_2_O molecules, which is conducive to the regeneration of hydroxyl groups. The pathway for the catalytic decomposition of O_3_ on NiFe-LDH catalysts at room temperature is a novel H-transfer redox mechanism with the H_2_O molecule as an intermediate (Fig. 8b, Eqs. (9)–(10)) [67]:

(9) $$O_{3}+2M-\text{OH}\to O_{2}+H_{2}O+2M-O$$

(10) $$2H_{2}O+4M-O\to O_{2}+4M-\text{OH}$$

Compared to NiAl-LDH, NiMn-LDH, CoFe-LDH and MgFe-LDH materials, the synergistic effect of Ni and Fe on NiFe-LDH catalysts induced the transfer of electrons from O atoms to H atoms, thus weakening the interactions between O atoms and H atoms, which facilitated hydrogen transfer reactions at room temperature (Fig. 8c) [70]. Of all the O_3_ decomposition catalysts reported to date, the NiFe-LDH catalyst has the best activity and stability (Fig. 8d–e) [67]. Under a relative humidity of 65% and a space velocity of 840 L⋅g^-1^⋅h^-1^ (600,000 h^-1^), the NiFe-LDH catalyst exhibits an O_3_ conversion of over 95% during 22 consecutive days of O_3_ decomposition testing (Fig. 8e). In order to optimize the preparation of conventional monolithic catalysts and overcome the degradation of the catalytic performance due to the weak bond between the catalyst powders and the support, NiFe-LDH monolithic catalysts self-loaded on iron foam (IF) were developed by a simple one-step hydrothermal method [71]. The NiFe-LDH/IF monolithic catalyst showed an O_3_ conversion of 85% after 168 h under harsh conditions, demonstrating great potential for the decomposition of O_3_ in practical applications [71].

The catalytic decomposition of O_3_ at room temperature has been extensively studied. Only a few studies have focused on catalytic decomposition of O_3_ at low temperatures for O_3_ purification in the cabin of aircraft flying at high altitudes. Tang’s group found that the sheet-shaped α-MnO_2_ with reactive (001) facets exhibited 100% conversion for 60 ppm O_3_ even at −10 °C under a space velocity of 510 L g^−1^ h^−1^, which is due to abundant surface oxygen vacancies [75]. Wang’s group found that the mullite YMn_2_O_5_ catalyst had 100% conversion for 120 ppm O_3_ at −5 °C under a space velocity of 600 L g^−1^ h^−1^, which is attributed to the moderate Mn−O bonding strength [61].

Currently, there are few studies on the effect of other pollutants on the O_3_ decomposition performance of catalysts. Abdallah et al. [76] found that HNO_3_-treated Ce-modified MnO_2_ catalyst exhibited stable O_3_ conversion of 94% in the presence of N_2_O_5_/HNO_3_, which is attributed to the high density of acid sites and oxygen vacancies. Tang et al. [77] studied the effect of SO_2_/H_2_O on the O_3_ decomposition performance of MnO_2_, discovering that cactus-like MnO_2_ nanospheres had excellent tolerance to water vapor and SO_2_/H_2_O. These cactus-like MnO_2_ nanospheres could maintain O_3_ conversion of > 88% in high humidity and sulfur-containing conditions. In summary, catalysts with excellent resistance to nitrogen-containing co-pollutants and SO_2_ can be obtained by tuning the physical-chemical structure and properties of MnO_2_, which is an important area for future research. In order to improve the resistance of catalysts to poisoning and maintain their activity in complex gas mixtures, the sacrificial sites for other pollutants should be designed, or the oxidation degree of co-existing pollutants during the reaction should be regulated by constructing specific active sites.

The economic feasibility and scalability of the three main catalysts (MnO*_x_*, Ag/MnO*_x_* and LDHs) for O_3_ decomposition are discussed below. In terms of economic feasibility, MnO*_x_* and LDHs catalysts are more feasible than Ag/MnO*_x_* catalysts because they use abundant and inexpensive transition metals as the main active component, avoiding the use of noble metals and significantly reducing the cost of raw materials. From the point of view of scalability, MnO*_x_* catalysts are typically prepared by hydrothermal and coprecipitation methods. These methods offer a broad range of temperature, pH and reaction time, making them insensitive to slight fluctuations. The Ag/MnO*_x_* catalysts are prepared by the simple impregnation method, making them easy to manufacture on an industrial scale. LDH catalysts are prepared by coprecipitation methods. The preparation parameters of LDH catalysts are insensitive to slight fluctuations. LDH catalysts can be scaled up by conventional equipment in a chemical plant without the need for special equipment.

## 3 Perspectives

Many studies have been carried out in recent years to address the problems of catalysts for the decomposition of O_3,_ and significant progress has been made. However, the competitive adsorption of H_2_O and O_3_ molecules is still a problem to be faced for most catalysts, including MnO*_x_* catalysts, Ag-based catalysts, and LDH catalysts. The problem can be solved either by inhibiting the occupation of active sites by H_2_O molecules to weaken the competitive adsorption of H_2_O and O_3_ molecules or by accelerating the activation of H_2_O molecules to facilitate the efficient participation of H_2_O molecules in the O_3_ decomposition. In addition, the desorption of the intermediate oxygen species is the key rate-limiting step on MnO*_x_* and Ag-based catalysts, so how to accelerate the capture of electrons from the intermediate oxygen species by the active sites is the key to improving the O_3_ decomposition activity and stability of the catalysts. The rate-limiting step in O_3_ decomposition on LDH catalysts is the activation of the intermediate product H_2_O molecules to form hydroxyl groups, so how to accelerate the activation of H_2_O molecules at the active sites is the key to improving the O_3_ decomposition activity and stability of LDH catalysts.

The key to regulating reaction processes on a catalyst is to regulate the physicochemical structures and properties of the catalyst, and significant progress has been made in this area. However, there is still a need for more precise tuning for specific catalysts. For manganese oxides, current studies mainly focus on the regulation of the number of oxygen vacancies. However, there are fewer studies on the regulation of the types and physicochemical properties of oxygen vacancies on manganese oxides, and a reliable theoretical system has not yet been developed. Therefore, more attention should be devoted to the preparation and microstructural characterization of manganese oxides. For Ag-based catalysts, the Ag sites with efficient activity of O_3_ decomposition still need to be investigated in detail, which first requires a more precise modulation of the Ag species. For LDH catalysts, the roles of each part of the LDH structure (interlayer hydroxyl groups, metal elements on the lamellae, and interlayer anions) during the O_3_ decomposition and the influences of these factors on the O_3_ decomposition performance have been initially clarified, but the precise regulation of the O—H bond strength of the active sites (hydroxyl groups) is lacking, which should be strengthened in the subsequent studies. In addition, new types of catalysts with superior O_3_ decomposition activity under humid gas, such as MOFs and covalent organic frameworks (COFs), have been successfully developed. However, the roles of specific parts of the MOF or COF structure (organic ligands, metal sites and their spatial arrangement) during the O_3_ decomposition and the influences of these factors on the O_3_ decomposition performance have not been clarified and should be addressed in follow-up studies.

In addition to humidity, the O_3_ decomposition performance of the catalyst will be affected by co-existing pollutants (SO_2_, NO*_x_*, CO, VOCs, etc.) in the actual application scenarios. The physicochemical properties of the co-existing pollutants, together with the type of active sites and the reaction mechanism on the catalysts, determine the influence mechanism of the co-existing pollutants on the O_3_ decomposition performance of the catalysts, which may be as follows: the competitive adsorption of the co-existing pollutants and O_3_ molecules on the active sites; the accumulation of the reaction products of the co-existing pollutants on the active sites; the influence of the co-existing pollutants on the pathway of O_3_ decomposition on the active sites. There are fewer studies on the effects of co-existing pollutants on the O_3_ decomposition performance of the catalysts, and the O_3_ decomposition performance of the catalysts should be further optimized in the future after determining the influence mechanisms of co-existing pollutants on the O_3_ decomposition performance of the catalysts. Besides, the catalysts used in aircraft cabins must withstand low temperatures of around −40 °C. Although Pd-based monolithic catalysts have been used in aircraft, they are expensive [11,14]. Current studies on the decomposition of O_3_ by MnO*_x_*, Ag-based, and LDH catalysts mainly focus on room temperature. The O_3_ decomposition performance of the MnO*_x_*, Ag-based, and LDH catalysts at low temperature should be studied as potential alternatives to Pd-based catalysts. In the future, other catalysts with efficient and stable activity at low temperatures should also be developed. Furthermore, the monolithic catalysts for real application must have excellent O_3_ decomposition performance at ultra-high space velocities [78,79], which can be achieved by regulating the active components, ceramic honeycomb supports, additives and binders.

## CRediT authorship contribution statement

**Xiaotong Li:** Writing – original draft, Visualization, Investigation, Funding acquisition, Data curation, Conceptualization. **Zhisheng Wang:** Writing – review & editing. **Biwu Chu:** Writing – review & editing. **Jinzhu Ma:** Writing – review & editing, Supervision, Resources, Project administration, Funding acquisition, Conceptualization. **Hong He:** Writing – review & editing, Supervision, Resources, Project administration, Funding acquisition, Conceptualization.

## Declaration of competing interest

The authors declare that they have no conflicts of interest in this work.
